# Association between Serum Phospholipid Fatty Acid Levels and Adiposity among Lebanese Adults: A Cross-Sectional Study

**DOI:** 10.3390/nu10101371

**Published:** 2018-09-25

**Authors:** Sahar G. Yammine, Farah Naja, Hani Tamim, Mona Nasrallah, Carine Biessy, Elom K. Aglago, Michèle Matta, Isabelle Romieu, Marc J. Gunter, Lara Nasreddine, Véronique Chajès

**Affiliations:** 1 Nutrition and Metabolism, International Agency for Research on Cancer, 69373 Lyon, France; yammines@students.iarc.fr (S.G.Y.); biessyc@iarc.fr (C.B.); AglagoE@fellows.iarc.fr (E.K.A.); michele.matta.92@gmail.com (M.M.); iromieu@gmail.com (I.R.); gunterm@iarc.fr (M.J.G.); 2 Nutritrion and Food Sciences Department, Faculty of Agricultural and Food Sciences, American University of Beirut, Beirut 1107 2020, Lebanon; fn14@aub.edu.lb (F.N.); htamim@aub.edu.lb (H.T.); mn36@aub.edu.lb (M.N.); ln10@aub.edu.lb (L.N.)

**Keywords:** nutrition, fatty acids, endogenous lipogenesis, obesity, epidemiology, low-to-middle income countries

## Abstract

There have been increases in the incidence of obesity in Lebanon over the past few decades. Fatty acid intake and metabolism have been postulated to influence obesity, but few epidemiological studies have been conducted. The aim of this study was to investigate the correlation between serum fatty acid levels and indicators of obesity in a cross-sectional study nested within a cohort of 501 Lebanese adults residing in Greater Beirut. A total of 395 available serum samples (129 men, 266 women) were profiled for phospholipid fatty acid composition. Spearman correlation coefficients adjusted for relevant confounders and corrected for multiple testing were calculated between serum fatty acids, desaturation indices, and indicators of adiposity (body mass index (BMI) and waist). BMI was significantly positively correlated with saturated fatty acids in men (r = 0.40, *p* < 0.0001, q < 0.0001) and women (r = 0.33, *p* < 0.0001, q < 0.0001). BMI was significantly positively correlated with monounsaturated fatty acid palmitoleic acid in women (r = 0.15, *p* = 0.01, q = 0.03). This study suggests that high blood levels of some saturated fatty acids and the monounsaturated fatty acid palmitoleic acid, likely derived from both dietary intakes of saturated fatty acids and endogenous lipogenesis, may have been associated with adiposity in the Lebanese population. The causality of these associations needs to be explored in experimental settings.

## 1. Introduction

The global prevalence of overweight adults in the world population has markedly increased from 24.6% in 1980 to 39% in 2016 [1,2]. Over the same period, the prevalence of obesity has nearly tripled worldwide from 6.4% to 13.0% [1,2]. In many countries, these changes have impacted the incidence of major non-communicable diseases including heart diseases, type 2 diabetes, and cancer [3]. In the eastern Mediterranean region, obesity rates in the adult population have reached high levels, exceeding at times those reported from developed countries such as the USA and Europe [4,5], with roughly one fifth of the adults in the region considered as obese [6]. Moreover, this increase in obesity rates has occurred in a short timeframe in the Middle East and is continuing to escalate [4,5]. In Lebanon, available data suggests that the prevalence of obesity increased significantly between the years 1997 and 2009 among adults aged 20 years and above (17.4% in year 1997 versus 28.2% in year 2009) [5].

The global expansion in obesity is predominantly attributed to changes in the obesogenic environment, characterized by (i) an upsurge in dietary energy intake, (ii) a higher consumption of added monosaccharides and of saturated and *trans* fatty acids, and (iii) an exceptional shift in energy expenditure patterns tilted towards a decrease in physical activity and an increase in sedentary behaviors [7]. Fat metabolism and dietary fatty acids have been postulated to affect obesity, estrogen levels, insulin resistance, and inflammation [8,9]. Several epidemiological studies have examined the relationship between dietary fatty acids estimated through dietary questionnaires and obesity, but the evidence remains inconclusive. Melanson et al. summarized interventional, prospective cohorts and cross-sectional studies investigating the associations between intakes of saturated acids (SFAs), monounsaturated acids (MUFAs), industrially-produced *trans* fatty acids (iTFAs), polyunsaturated fatty acids (PUFAs), and risk of obesity. The authors of this review reported that there is inconclusive evidence regarding the associations between the amount and types of fat intake and obesity. This review also underscored the inconsistencies in the literature and highlighted the limitations of dietary assessment methods as potential reasons underlying these inconsistencies [10]. In fact, whether collected using dietary recalls or records methodologies, dietary intake estimations have inherent biases and errors that affect their accuracy.

As a complementary tool to information based on dietary assessment methods, the measurement of serum or plasma fatty acids might provide a more objective estimation to enable a better understanding of their impact on obesity. Hence, some epidemiological studies based on the use of circulating fatty acids have consistently reported a positive association between adipose tissue or circulating palmitoleic acid, DI_16_, and obesity [11,12,13,14,15]. Furthermore, a prospective study conducted within the European Prospective Investigation on Cancer (EPIC) reported an increased risk of weight gain during the follow-up associated with increasing levels of plasma phospholipid industrial *trans* elaidic acid [16], suggesting that iTFAs might increase adiposity. Further epidemiological studies based on biomarkers of fatty acids are needed to clarify the association between fatty acids and obesity.

Based on this set of data, we hypothesized that a high intake of iTFAs, along with an increased endogenous synthesis of MUFAs, may increase adiposity. Building on this framework, the aims of this study are to characterize the serum phospholipid profile in a cross-sectional study designed among Lebanese adults residing in the Greater Beirut area, and to determine the correlation between fatty acids, as biomarkers of dietary exposure and endogenous fatty acid metabolism, and obesity indicators. 

## 2. Materials and Methods

### 2.1. Study Population and Recruitment 

The target population constituted of Lebanese adults (>18 years) residing in the Greater Beirut area. The study sample for this study was drawn from an earlier community-based survey of a representative sample of Lebanese adults living in Greater Beirut area selected using a multistage stratified probability sampling frame. Details on the sampling used in this study are described elsewhere [17]. Pregnant women, patients on dialysis, and other vulnerable groups (mentally disabled patients) were excluded. Furthermore, given that the original survey aimed to examine exposure to bisphenol A (BPA) among adults residing in Beirut, participants working in plastic or other chemical companies were excluded as they may have been occupationally exposed to bisphenol A. Of the total 501 study participants, 395 participants consented to the use of their serum samples for future studies and hence were included in the current study.

### 2.2. Data Collection

In a face-to-face interview, trained interviewers completed a detailed data collection form for each subject. This questionnaire included information pertaining to the participants’ medical history (all diseases that are associated with BPA and medications), diet through a food frequency questionnaire (FFQ) as well as lifestyle habits (smoking, alcohol, coffee, and physical activity), and socio-demographic information (age, gender, residence and previous travel, education, occupation, and income), physical exams (anthropometric data, weight, height, BMI (body mass index), waist circumference, and blood pressure), and a collection of urine and blood samples. Blood samples were collected after an overnight fast. The FFQ was an 80-item, semi-quantitative questionnaire, referring to subjects’ dietary intake 12 months prior to the interview [17]. A tetrapolar single-frequency (330 μA at 100 kHz) electrical bioimpedance analyzer was used to measure body composition. All interviews, physical examinations, and collection of biological samples were performed at the Nutrition and Food Sciences department, American University of Beirut (AUB). 

### 2.3. Ethical Considerations

The protocol of the original survey was approved by the Institutional Review Board (IRB) at AUB. This study was approved by both the AUB IRB and the Ethical Committee of the International Agency for Research on Cancer.

### 2.4. Analysis of Serum Phospholipid Fatty Acids 

For the purpose of this study, the 395 available blood samples were shipped to the International Agency for Research on Cancer (IARC) and stored at −80 °C until analyzed. As previously described [18], total lipids were extracted from serum samples, the phospholipid fraction was purified by adsorption chromatography, and Methyl-Prep II was used for the thansmethylation of fatty acids into fatty acid methyl esters. Fatty acid methyl esters were eluted on a gas chromatograph 7890A (Agilent Technologies, Santa Clara, CA, USA). Select for Fame capillary columns and specific for TFA separation were used for the separation of fatty acid methyl esters. Fatty acids are expressed as percent of total fatty acids and as absolute concentrations in serum (µmol/liter) based on the quantity of l-a-phosphatidylcholine-dimyristoyl-d_54_ used as an internal standard. Overall (intra-batch and inter-batch) coefficients of variation (CVs) for fatty acids, which were calculated using two serum samples as quality controls added to each batch, ranged from 0.290% for large peaks, such as palmitic acid, to 9.340% for the smallest peaks, such as CLA. Overall CVs were 0.850 for saturated fatty acids, 0.291 for total monounsaturated fatty acids, 0.522 for industrial *trans* fatty acids, 0.312 for n-3 polyunsaturated fatty acids, and 0.974 for n-6 polyunsaturated fatty acids.

Using values for 60 individual fatty acids, the percentage and amounts of the following groups were calculated: SFAs, *cis*-MUFAs, ruminant *trans* fatty acids (rTFAs), iTFAs, *cis*-n-6 PUFAs, long-chain n-6 PUFAs, n-3 PUFAs, and long-chain n-3 PUFAs. We calculated the ratio of long-chain n-6/long-chain n-3 PUFAs. The desaturation indexes, as the ratio of product to substrate, either oleic acid to stearic acid (DI_18_) or the ratio of palmitoleic acid to palmitic acid (DI_16_), as biomarkers of endogenous lipogenesis, were also determined [19].

### 2.5. Statistical Analyses

The sociodemographic and lifestyle characteristics, as well as dietary intake of the study population, are represented in terms of frequencies for the categorical variables and means ± standard deviations (SD) for the continuous variables. Fatty acids expressed in percentage of total fatty acids and in amounts were used for the statistical analysis. Fatty acid values were log-transformed and geometric means with 95% confidence interval (CI) were provided for the analysis. As first screening, fatty acids or desaturation indices were correlated with obesity indicators (BMI and waist circumference) using a partial Spearman test. Linear regression using the least squares method was applied to test for potential non-linear associations between BMI and Waist with each of the fatty acids in turn. The model tested for both a linear and a squared term for the parameters. For the fatty acids, which were significantly correlated with BMI and Waist, we also assessed the assumptions of linearity using the linearity test, which showed that the analyzed correlations demonstrated a linear distribution; hence, we proceeded with statistical tests of linear correlation. Statistical analyses were also run in relation to percentage of body fat (data not shown). Adjustments were performed for the following factors: age (continuous variable), menopausal status in women (pre- and postmenopause), physical activity (total MET minutes/week), smoking status (non-smokers, current smokers, and ex-smokers), education (none, incomplete primary, complete primary, complete secondary, and complete high school), alcohol consumption (g/day), energy intake (kcal/day), and analytical batch. When considering the ratio of fatty acids (DI, n-6/n-3 PUFAs), fatty acids included in the ratio were further included in the statistical model. The coefficient of correlation (r) and the *p*-value were provided. Due to the number of tests performed, q-values were calculated by transforming the *p*-values for multiple comparisons using the false discovery rate of the Benjamini–Hochberg procedure [20]. Statistical analyses were performed using STATA version 14.1 (StataCorp, College Station, TX, USA) and R (R Foundation for Statistical Computing version 3.0.2, Vienna, Austria). A *p*-value ≤ 0.05 was used to indicate significance for all tests. 

## 3. Results

### 3.1. Subjects Characteristics

General characteristics of the study participants are presented in Table 1, separately for men and women, the latter constituting approximately 2/3 of the participants. Overall, the studied population is characterized by a high BMI with a high percentage of obese subjects, a high percentage of smokers among men and women, with a significantly higher total energy intake in men than in women (Table 1).

### 3.2. Serum Phospholipid Fatty Acid Composition

Serum phospholipid fatty acids, expressed as a percentage of total fatty acids, are indicated in Table 2, separately for men and for women. Individual fatty acids are grouped by family (SFAs, MUFAs, rTFAs, iTFAs, and n-6 and n-3 PUFAs) and by conformation (*trans* and *cis*). 

Palmitic acid (16:0) and linoleic acid (18:2n-6*cis*) were the most abundant fatty acids in men and women in this population, accounting for the high percentages of total SFAs and total n-6 PUFAs, respectively (Table 2). The percentage of n-3 PUFAs was substantially lower than n-6 PUFAs, exhibiting a high ratio n-6/n-3 PUFA of 10.46 in men and 10.94 in women. Among TFA isomers, iTFAs represented 0.50% in men and 0.48% in women, while rTFAs represented 0.15% in men and in women. Total MUFAs, iTFAs, total TFAs, and n-3 PUFAs were significantly higher in men than in women, while total SFAs was higher in women compared to men. The odd-chain fatty acids, pentadecanoic acid (15:0) and heptadecanoic acid (17:0), derived from dairy foods, were higher in women compared to men.

### 3.3. Correlation between Serum Fatty Acids and Indicators of Obesity

Table 3 and Table 4 show the Spearman coefficients of correlation between fatty acid families and indicators of obesity, BMI, and waist circumference. 

BMI was significantly positively correlated with total SFAs in both men (r = 0.40, *p* < 0.0001, q < 0.0001) and women (r = 0.33, *p* < 0.0001, q < 0.0001) (*p* of heterogeneity = 0.035). In terms of individual SFA, no significant correlation was found between BMI or waist and the odd-chain fatty acids, pentadecanoic acid (15:0) and heptadecanoic acid (17:0). Similar trends were found with waist circumference (Table 4) and with percentage of body fat (data not shown).

In women particularly, BMI was significantly positively correlated with MUFA palmitoleic acid (r = 0.15, *p* = 0.01, q = 0.03). A weak positive association was also reported with the DI_16_ (r = 0.13, *p* = 0.03, q = 0.07), but that did not withstand correction for multiple testing. Further adjustment for palmitic acid and palmitoleic acid did not change the correlation (data not shown). In contrast, a negative correlation was found in women between BMI and total iTFAs (r = −0.17, *p* = 0.007, q = 0.03). When we distinguished individual TFA isomers, we found differential correlations with BMI according to gender, with elaidic acid (r = −0.14, *p* = 0.02, q = 0.05), and *trans* linoleic acid (r = −0.15, *p* = 0.01, q = 0.03) showing significant inverse correlations in women, while *trans* isomers of α-linolenic acid showed a positive trend in men (r = 0.22, *p* = 0.01, q = 0.10). Similar trends were found with waist circumference (Table 4) and with percentage of body fat (data not shown).

No significant correlation was found between n-6 PUFAs, n-3 PUFAs, or the ratio n-6/n-3 PUFAs and BMI, waist circumference, and percentage of body fat (data not shown). When considering the ratio, further adjustment for n-6 and n-3 PUFAs did not change the correlation (data not shown). Divergent correlations according to gender were found between individual n-6 and n-3 PUFAs and BMI. In men, n-3 α-linolenic acid, the essential fatty acid of the n-3 family, tended to be positively correlated with obesity (r = 0.18, *p* = 0.04, q = 0.24), while a negative trend was found for long-chain n-3 docosahexaenoic acid (DHA) (r = −0.24, *p* = 0.009, q = 0.10). In women, n-6 γ-linoleic acid (r = 0.17, *p* = 0.006, q = 0.03) and n-3 eicosapentanoic acid (EPA) (r = 0.14, *p* = 0.02, q = 0.05) were positively correlated with BMI. 

Similar correlations with BMI, waist circumference, and percentage of body fat were found when fatty acids were expressed in amounts (data not shown).

All individual fatty acids which were significantly correlated with indicators of obesity showed a significant linear relationship with BMI and waist. A non-statistically significant linear relationship was found between BMI or waist and all other individual fatty acids, such as pentadecanoic acid (15:0), heptadecanoic acid (17:0), and most of the n-6 and n-3 PUFAs (data not shown).

## 4. Discussion

This is the first population-based study reporting serum phospholipid fatty acid profiles in a Lebanese population and their correlations with indicators of adiposity. We found that total levels of SFAs were positively correlated with BMI in both men and women. Palmitoleic acid and DI_16_, as biomarkers of endogenous lipogenesis, were positively correlated with BMI, particularly in women. Divergent correlations were reported between individual *trans* fatty acids, n-6 and n-3 PUFAs, and BMI. Similar trends were found in relation to waist circumference and percentage of body fat. These findings suggest that different subtypes of fatty acids may differentially impact obesity. Further, we identified a specific fatty acid profile in this Lebanese population compared to other populations.

The measurement of serum phospholipid fatty acids is a complementary tool to estimate dietary fatty acid intake through dietary assessment methods. Serum or plasma phospholipid fatty acids represent specific biomarkers of past dietary intakes (weeks to months) of fatty acids that cannot be endogenously synthesized, such as PUFAs and iTFAs [19,21,22]. In contrast, weak associations were found between dietary intakes of SFAs and MUFAs and their respective levels in plasma phospholipid, likely because of endogenous synthesis of these fatty acids [19,22]. A significant positive association was found between plasma MUFAs or DI_16_ and dietary intakes of SFA, suggesting that blood phospholipid MUFAs are biomarkers of dietary SFA and endogenous lipogenesis [19,22]. Thus, SFA and MUFA levels in blood phospholipid fraction among free-living individuals are likely to be markers of both dietary intake and de novo lipogenesis [19,23]. 

It is challenging to determine whether the distribution of various fatty acids in a given population is low or high due to a lack of appropriate reference values. As an alternative, we compared serum phospholipid fatty acid profiles in Lebanese adults to those reported in participants from the Mediterranean regions (Athens, Spain, and Ragusa/Naples) in the European Prospective Investigation into Cancer and Nutrition (EPIC) cohort, based on data measured in our laboratory using the same methodology [22]. Compared to Mediterranean European adults, the fatty acid profile of Lebanese adults is markedly different, particularly regarding PUFA levels. The most prominent difference in Lebanon is a higher level of n-6 PUFA (44.23% in Lebanon versus 38.95% in Mediterranean regions in the EPIC study), presumably originating from vegetable oils, and low levels of n-3 PUFA derived from fish (4.10% vs. 7.57%). Accordingly, the ratio of n-6/n-3 PUFA in the Lebanese population is much higher than in Mediterranean regions of EPIC (10.78 vs. 5.15) [22]. Levels of MUFA (11.34% vs. 13.71%) are lower in Lebanese adults than in Mediterranean European adults. In contrast, total levels of SFA in Lebanon individuals were found comparable to those reported in Mediterranean regions of EPIC (39.25% of total fatty acids in Lebanon versus vs. 39.76% in Mediterranean regions of EPIC) as well as the levels of *trans* elaidic acid (0.14% vs. 0.15%). Even if the two study populations differ on different characteristics, for example mean age at recruitment (47.3 years in the Lebanese study vs. 53.9 years in EPIC), mean BMI at recruitment (29.6 in the Lebanese study vs. 25.5 in the EPIC study) or date at blood collection (2014 in the Lebanese cohort vs. 1992–1998 for the EPIC study), we previously showed that geographic region appeared to be the strongest determinant factor explaining variability in blood levels of fatty acids [22].

We found that total SFAs were significantly positively correlated with BMI and waist circumference, which was somewhat stronger in men than in women. Similar trends between total SFAs and BMI have been reported in a cross-sectional analysis among Mexican women [24]. In agreement with our findings, a review of epidemiological studies and clinical trials described that SFA consumption led to increased body adiposity [25]. Similarly, SFA intake has been linked to obesity and specifically to abdominal fat accumulation among women enrolled in the Nurses’ Health study [26] and among U.S. men in another prospective study [25]. We found that the positive correlations between total SFAs and BMI or waist circumference are likely to be driven by palmitic acid in men and stearic acid in women. In contrast, among odd-chain saturated fatty acids originating from dairy foods, heptadecanoic acid showed a non-significant inverse association with BMI and waist circumference in women. These data suggest that individual SFAs may have differential effects on adiposity depending on their dietary sources and endogenous synthesis. Furthermore, we found that palmitoleic acid and DI_16_, as biomarkers of endogenous lipogenesis, were positively correlated with BMI, particularly in women. Our data further suggest that an increased endogenous synthesis of palmitoleic acid may increase adiposity. In agreement with our findings, some epidemiological studies have consistently reported a positive association between adipose tissue or circulating palmitoleic acid, DI_16_ and obesity [11,12,13,14,15]. Furthermore, an epidemiological study among Japanese employees indicated that high levels of serum palmitoleic acid levels led to increased concentrations of C-peptide, insulin resistance, and inflammation [27], known factors involved in obesity. Our data suggest that increased endogenous synthesis of palmitoleic acid may increase adiposity. Further studies are needed to explore the causality of the association between increased synthesis of palmitoleic acid and obesity, and whether this effect might be mediated by insulin resistance and inflammation. 

Inconsistent trends were found in this study between levels of total iTFAs, individual iTFA isomers, and BMI or waist circumference among women, albeit these correlations were weak. When we distinguished individual TFA isomers, we found differential correlations with BMI according to gender, with elaidic acid and *trans* linoleic acid showing significant inverse correlations in women, while *trans* isomers of α-linolenic acid showing a positive trend in men. Similar to our finding, a cross sectional analysis among Costa Rican adults reported divergent associations between TFA isomers and adiposity [28]. In particular, negative associations between *trans* isomers of 18:1 (as the sum of 18:1n-7, 18:1n-9, and 18:1n-11) measured in adipose tissue and all measures of adiposity (visceral and subcutaneous adiposity) were reported [28]. This inverse association was explained by the relatively low consumption of *trans* isomers of 18:1 in Costa Rica [28]. Also, no clear association was observed between plasma phospholipid levels of total *trans* fatty acids (as the sum of 16:1, 18:1, and 18:2) and baseline BMI or BMI changes (during 10 years of follow-up) in a cross-sectional and longitudinal study with available repeated measurements within the American Multi-Ethnic Study of Atherosclerosis (MESA) cohort [29]. In contrast, high baseline blood levels of iTFA elaidic acid have been associated with an increased risk of weight gain during a 5-year follow-up in the European EPIC cohort [16]. In agreement with this finding, a significant positive association between levels of total *trans* 18:1 measured in erythrocyte membranes and weight gain was reported in American women during a 10.4-year follow-up [30]. Data from experimental models suggested that iTFA may induce obesity. A long-term intervention study on primates reported an increase of body weight in animals receiving an iTFA diet compared to those receiving *cis*-fatty acids [31,32]. Another study showed that a diet high in *trans* fat induces insulin resistance pathway and obesity [33]. The association between iTFA and obesity still remains unclear and needs further investigation in prospective settings. 

N-6 and n-3 PUFAs may have divergent effects on the development of obesity through their differential effect on inflammation [34]. In our study population, divergent correlations according to gender were reported between individual n-6 and n-3 PUFAs and obesity. In men, n-3 α-linolenic acid, the essential fatty acid of the n-3 family, tended to be positively correlated with obesity, while a negative trend was found for long-chain n-3 docosahexaenoic acid (DHA). In women, n-6 γ-linoleic acid and n-3 eicosapentanoic acid (EPA) were positively correlated with obesity. A similar positive trend between EPA and BMI was reported in a cross-sectional study among Mexican women [17]. Similarly, a positive association was found between levels of EPA in blood cholesterol esters and abdominal obesity in Swedish women but not in men [15]. Although the ratio n-6/n-3 PUFAs in our study is high (10.78), no significant correlation was found with indicators of obesity. In contrast, n-6/n-3 PUFAs was associated with an increased risk of weight gain in the Women Health Initiative (WHI) study [30], despite the fact that this ratio was much lower in this population (4.68) compared to the ratio reported in the present study. This discrepancy between studies might be the consequence of the design, prospective versus cross-sectional, of the levels of PUFAs reported in each population, and of the sample size of each study. Further studies with a prospective design are needed to investigate the association between n-6 and n-3 PUFAs, as well as the ratio n-6/n-3 PUFAs, and obesity.

This study has characterized the serum phospholipid fatty acid profile in a Lebanese population and highlighted important differences with a European population living in Mediterranean regions. However, the findings of this study are limited by the cross-sectional nature of the analysis and these data need to be replicated in a prospective setting. 

## 5. Conclusions

In conclusion, this study suggests that high blood levels of some SFAs and MUFA palmitoleic acid, likely to derive from dietary intake of SFA and increased endogenous lipogenesis, is correlated with increased adiposity in the Lebanese population. The causality of these associations remains to be investigated. Reducing SFA intakes could potentially offer a public health strategy for reducing BMI. In addition to being the first of its kind in the Middle East, this report provides a timely framework to examine biomarkers and health effects in a region currently undergoing a nutritional transition. 

## Figures and Tables

**Table 1:** Baseline characteristics of the studied population.

	Mean ± SD or *N* (%) Total *N* = 395	Mean ± SD or *N* (%) Men *N* = 129	Mean ± SD or *N* (%) Women *N* = 266	*p* Value ^a^
Age, years	44.5 ± 15.3	38.8 ± 16.3	47.3 ± 14.0	<0.0001
Anthropometry				
Weight, kg	75.2 ± 15.5	81.2 ± 15.5	72.2 ± 15.6	<0.0001
Height, cm	161.5 ± 9.8	172.2 ± 6.5	156.3 ± 6.4	<0.0001
Body-fat, kg	28.1 ± 11.4	22.6 ± 10.9	30.74± 10.7	<0.0001
BMI, kg/m^2^	28.9 ± 5.7	27.4 ± 5.0	29.6 ± 5.9	0.0003
Percent body-fat, %	36.8 ± 10.9	26.7 ± 8.8	41.6 ± 8.1	<0.0001
Waist circumference, cm	94.5 ± 15.3	96.1 ± 12.7	93.8 ± 16.4	NS
BMI cut points, *N* (%)				0.01
Underweight and normoweight *N* (<25 kg/m^2^)	103 (26.1%)	42 (32.6%)	61 (22.9%)	
Overweight (25–29.99 kg/m^2^)	132 (33.4%)	48 (37.2%)	84 (31.6%)	
Obese (≥30 kg/m^2^)	160 (40.5%)	39 (30.2%)	121 (45.5%)	
Percent body-fat cut points, *N* (%)				<0.0001
Normal ≤25% for men ≤35% for women		58 (44.9%)	51 (19.2%)	
Obese >25% for men >35% for women		71 (55.1%)	215 (80.8%)	
Waist circumference cut points, *N* (%)				<0.0001
Normal <94 cm for men <80 cm for women		53 (41.1%)	50 (18.8%)	
Increased risk of metabolic complications (94–102 cm) for men (80–88 cm) for women		39 (30.2%)	53 (19.9%)	
Substantially increased risk for metabolic complications >102 cm for men >88 cm for women		37 (28.7%)	163 (91.3%)	
Menopausal status, N (%)				
Pre-menopause			142 (53.4%)	
Post-menopause			124 (46.6%)	
Lifestyle factors				
Physical activity, total Mets/week	1731.9 ± 2129.7	1805.9 ± 2270.4	1696.0 ± 2061.5	NS
Smoking, N (% of current smokers)	258 (65.3%)	99 (76.7%)	159 (59.8%)	0.001
Nutritional factors				
Energy intake, kcal/day	3361.2 ± 1969.9	4839.4 ± 2411.7	2644.3 ± 1174.9	<0.0001
Protein intake, g/day	109.9 ± 73.9	160.5 ± 83.4	85.4 ± 54.1	<0.0001
Percent of total energy intake	13.2	13.6	13.0	NS
Carbohydrate intake, g/day	415.9 ± 242.6	583.8 ± 301.1	334.5 ± 152.9	<0.0001
Percent of total energy intake	50.5	49.2	51.2	0.03
Total fat intake, g/day	138.7 ± 88.9	195.1 ± 109.4	111.4 ± 60.8	<0.0001
Percent of total energy intake	36.8	35.8	37.3	NS
Alcohol intake, g/day	7.4 ± 37.8	22.4 ± 63.8	0.18 ± 1.1	<0.0001
Percent of total energy intake	0.86	2.53	0.05	<0.0001

^a^ Independent-sample *t*-test or chi-square test. BMI: Body Mass Index; NS: non-significant; SD: standard deviation.

**Table 2:** Serum Phospholipid fatty acids in the population.

	Mean (95% CI) ^a^ *N* = 395	Mean (95% CI) ^a^ Men *N* = 129	Mean (95% CI) ^a^ Women *N* = 266	*p* Value ^b^
Saturated fatty acids (SFAs)				
14:0 (myristic acid)	0.19 (0.18; 0.20)	0.17 (0.16; 0.19)	0.20 (0.18; 0.21)	0.04
15:0 (pentanoic acid)	0.14 (0.13; 0.143)	0.13 (0.11; 0.14)	0.14 (0.13; 0.15)	0.004
16:0 (palmitic acid)	23.16 (22.86; 23.46)	22.68 (22.01;23.36)	23.40 (23.09; 23.70)	0.03
17:0 (heptadecanoic acid)	0.41 (0.40; 0.42)	0.39 (0.38; 0.41)	0.41 (0.40; 0.42)	0.02
18:0 (stearic acid)	15.13 (14.99;15.26)	14.98 (14.77; 15.19)	15.20 (15.02; 15.37)	NS
Monounsaturated fatty acids (MUFAs)				
*cis*-MUFAs				
16:1n-7 (palmitoleic acid)	0.59 (0.57; 0.61)	0.56 (0.53; 0.59)	0.61 (0.58; 0.63)	0.03
18:1n-5	0.03 (0.029; 0.034)	0.037 (0.034; 0.04)	0.033 (0.031; 0.035)	NS
18:1n-7 (*cis*-vaccenic acid)	1.24 (1.22; 1.26)	1.23 (1.20; 1.27)	1.25 (1.22; 1.27)	NS
18:1n-9 (oleic acid)	9.12 (8.99; 9.25)	9.40 (9.17; 9.64)	8.99 (8.84; 9.15)	0.003
*trans*-MUFA*s*				
16:1n-7/9 (palmitelaidic acid)	0.22 (0.21; 0.23)	0.22 (0.21;0.23)	0.22 (0.21; 0.23)	NS
18:1n-9/12 (elaidic acid)	0.14 (0.13; 0.15)	0.14 (0.13; 0.15)	0.14 (0.129; 0.145)	NS
18:1n-7 (vaccenic acid)	0.03 (0.02; 0.04)	0.07 (0.06; 0.08)	0.065 (0.061; 0.069)	NS
Polyunsaturated fatty acids (PUFAs)				
*cis* n-6 PUFAs				
18:2n-6 (linoleic acid)	24.42 (24.12; 24.72)	24.89 (24.32; 25.48)	24.19 (23.84; 24.54)	0.03
18:3n-6 (γ-linolenic acid)	0.16 (0.15; 0.17)	0.16 (0.14;0.17)	0.16 (0.15; 0.17)	NS
20:3n-6 (di-homo-γ-linolenic acid)	4.12 (4.02; 4.22)	3.86 (3.69; 4.03)	4.25 (4.12; 4.37)	0.0003
20:4n-6 (arachidonic acid)	13.49 (13.25; 13.74)	13.39 (12.94; 13.87)	13.53 (13.25; 13.82)	NS
22:4n-6 (adrenic acid)	0.60 (0.59; 0.61)	0.61 (0.59; 0.63)	0.59 (0.58; 0.61)	NS
22:5n-6 (osbond acid)	0.47 (0.46; 0.49)	0.44 (0.42;0.47)	0.49 (0.47;0.51)	0.003
*Trans*-n-6 PUFAs				
Conjugated linoleic acid (CLA)	0.078 (0.07;0.08)	0.077 (0.071; 0.083)	0.078 (0.074; 0.082)	NS
18:2ct, 18:2tc, 18:2tt (*trans* linoleic acid)	0.89 (0.80; 0.90)	0.099 (0.092; 0.11)	0.081 (0.077; 0.085)	<0.0001
*cis-n-9 PUFA*				
20:3n-9 (mead acid)	0.095 (0.092; 0.098)	0.090 (0.084; 0.097)	0.096 (0.092; 0.10)	0.04
*cis-n-3 PUFA*				
18:3n-3ccc (α-linolenic acid)	0.11 (0.11; 0.12)	0.12 (0.11; 0.13)	0.11 (0.10; 0.12)	NS
20:5n-3 (eicosapentaenoic acid, EPA)	0.30 (0.29; 0.32)	0.32 (0.29; 0.36)	0.30 (0.28; 0.31)	NS
22:5n-3 (docosapentaenoic acid, DPA)	0.71 (0.69,0.73)	0.78 (0.74; 0.81)	0.68 (0.66;0.70)	<0.0001
22:6n-3 (docosahexaenoic acid, DHA)	2.84 (2.77; 2.91)	2.89 (2.76;3.03)	2.82 (2.73; 2.90)	NS
*Trans-n-3 PUFAs*				
18:3n-3cct, ctt, ttt (*trans* α-linolenic acid)	0.01 (0.008; 0.012)	0.01 (0.014; 0.017)	0.01 (0.015; 0.017)	NS
Groupings				
Total SFAs	39.25 (39.02; 39.47)	38.65 (38.16; 39.14)	39.54 (39.32; 39.77)	<0.0001
Total *cis*-MUFAs	11.34 (11.19; 11.48)	11.58 (11.33; 11.84)	11.22 (11.05; 11.39)	0.02
Total *trans* ruminant fatty acids	0.14 (0.13; 0.15)	0.15 (0.14; 0.16)	0.14 (0.13; 0.145)	NS
Total *trans* industrial fatty acids	0.49 (0.48; 0.50)	0.50 (0.48;0.52)	0.48 (0.47; 0.50)	0.007
Total *trans* fatty acids	0.64 (0.63;0.66)	0.66 (0.63;0.69)	0.63 (0.61; 0.65)	0.04
Total *cis* n-6 PUFAs	44.23 (44.00; 44.46)	44.34 (43.86; 44.83)	44.17 (43.92; 44.42)	NS
Total long-chain n-6 PUFAs	19.33 (19.07; 19.59)	18.93 (18.45; 19.43)	19.52 (19.22; 19.82)	0.04
Total *cis* n-3 PUFAs	4.10 (4.01; 4.19)	4.24 (4.07; 4.42)	4.03 (3.93; 4.13)	0.03
Total long-chain n-3 PUFAs	3.97 (3.89; 4.06)	4.11 (3.94;4.29)	3.91 (3.81; 4.01)	0.04
Long-chain n-6/n-3 PUFAs	4.86 (4.74; 4.97)	4.61 (4.41; 4.82)	4.99 (4.85; 5.13)	0.002
Ratio n-6/n-3 PUFAs	10.78 (10.53;11.04)	10.46 (10.00; 10.94)	10.94 (10.65; 11.25)	NS
Desaturation indexes				
Desaturation index_16_ (16:1n-7*cis*/16:0)	0.026 (0.025;0.026)	0.025 (0.024;0.026)	0.026 (0.025; 0.027)	NS
Desaturation index_18_ (18:1n-9*cis*/18:0)	0.60 (0.59; 0.61)	0.63 (0.61;0.65)	0.59 (0.58; 0.60)	0.003

**^a^** Fatty acids are expressed as a percentage of total fatty acids and represented as geometric means with 95% confidence intervals (CIs); ^b^ Independent-sample *t*-test.

**Table 3:** Partial Spearman ^a^ correlation between serum phospholipid fatty acids and BMI.

	Men (*N* = 129)			Women (*N* = 266)			
Fatty Acids (Percentage of Total Fatty Acids)	r	*p*	q ^b^	r	*p*	q ^b^	*p* of Heterogeneity
Saturated fatty acids (SFAs)							
Pentadecanoic acid (15:0)	0.17	0.06	0.26	-0.009	0.88	0.88	NS
Heptadecanoic acid (17:0)	−0.09	0.30	0.44	−0.11	0.07	0.12	NS
Palmitic acid (16:0)	0.19	0.03	0.22	0.04	0.53	0.69	NS
Stearic acid (18:0)	0.11	0.22	0.43	0.26	<0.0001	<0.001	NS
Total SFA ^c^	0.40	<0.0001	<0.001	0.33	<0.0001	<0.0001	0.03
*cis*-Monounsaturated fatty acids (MUFAs)							
Palmitoleic acid (16:1n-7,9)	0.18	0.049	0.24	0.15	0.01	0.03	NS
Oleic acid (18:1n-9)	−0.09	0.34	0.46	−0.20	0.001	0.006	NS
Total *cis*-MUFA ^d^	−0.12	0.19	0.43	−0.20	0.001	0.006	NS
n-6 polyunsaturated fatty acids (PUFAs)							
Linoleic acid (18:2n-6)	−0.07	0.44	0.55	−0.05	0.45	0.64	NS
γ-Linolenic acid (18:3n-6)	0.05	0.57	0.65	0.17	0.006	0.03	NS
Arachidonic acid (20:4n-6)	−0.10	0.26	0.43	0.03	0.66	0.75	NS
Total n-6 PUFAs ^e^	−0.09	0.29	0.44	−0.05	0.38	0.57	NS
Total long-chain n-6 PUFAs ^f^	−0.03	0.73	0.75	0.04	0.48	0.65	NS
*cis*-n-3 PUFAs							
α-linolenic acid (18:3n-3)	0.18	0.04	0.24	−0.12	0.06	0.11	0.02
Eicosapentaenoic acid (EPA, 20:5n-3)	0.09	0.31	0.44	0.14	0.02	0.05	NS
Docosahexaenoic acid (DHA, 22:6n-3)	−0.24	0.009	0.10	−0.03	0.61	0.75	NS
Total n-3 PUFAs ^g^	−0.14	0.12	0.35	−0.03	0.63	0.75	NS
Total long-chain n-3 PUFAs ^h^	−0.16	0.08	0.26	−0.02	0.74	0.79	NS
Industrial *trans* fatty acids (iTFAs)							
Palmitelaidic acid (16:1n-9)	0.11	0.23	0.43	−0.12	0.05	0.10	NS
Elaidic acid (18:1n-9/12)	−0.14	0.13	0.35	−0.14	0.02	0.05	NS
Linoleic acid (18:2tt, ct, tc)	−0.04	0.62	0.66	−0.15	0.01	0.03	NS
α-Linolenic acid (18:3n-3ctt, ttc)	0.22	0.01	0.10	0.07	0.24	0.40	NS
Total iTFAs	0.009	0.92	0.92	−0.17	0.007	0.03	NS
Ruminant *trans* fatty acids (rTFAs)							
Vaccenic acid (18:1n-7)	−0.16	0.07	0.26	−0.12	0.05	0.11	NS
Conjugated linoleic acids (CLAs, 9c-11t; 10t, 12c)	0.05	0.59	0.65	−0.06	0.32	0.51	NS
Total rTFAs	−0.05	0.54	0.65	−0.11	0.06	0.11	NS
Ratio							
n-6 PUFAs/n-3 PUFAs	0.10	0.26	0.43	0.018	0.77	0.79	NS
Long-chain n-6 PUFA/long-chain n-3 PUFAs	0.08	0.38	0.49	0.02	0.68	0.75	NS
Desaturation index_16_ (DI_16_, 16:1/16:0)	0.12	0.20	0.43	0.13	0.03	0.07	NS
Desaturation index_18_ (DI_18_, 18:1/18:0)	−0.11	0.22	0.43	−0.26	<0.001	<0.001	0.03

^a^ Models were adjusted for age, alcohol consumption, smoking, energy intake, education, physical activity, menopausal status in women, and batch of analysis. ^b^ Value for FDR (False Discovery Rate) correction. ^c^ Total SFA included 10:0, 12:0, 14:0, 15:0, 16:0, 17:0, 18:0, 20:0, 22:0, 24:0; ^d^ Total *cis*-MUFA included 14:1, 15:1, 16:1n-7,9, 17:1, 18:1n-5, 7, 9, 20:1, 22:1, 24:1; ^e^ Total n-6 PUFA included 18:2, 18:3, 20:2, 20:3, 20:4, 22:4, 22:5; ^f^ Total long-chain n-6 PUFA included 20:2, 20:3, 20:4, 22:4, 22:5; ^g^ Total n-3 PUFA included 18:3, 18:4, 20:4, 20:5, 22:5, 22:6; ^h^ Total long-chain n-3 PUFA included 20:4, 20:5, 22:5, 22:6.

**Table 4:** Partial Spearman ^a^ correlations between serum phospholipid fatty acids and waist circumference.

	Men (*n* = 129)			Women (*n* = 266)			
Fatty acids (Percentage of Total Fatty Acids)	r	*p*	q ^b^	r	*p*	q ^b^	*p* of Heterogeneity
Saturated fatty acids (SFAs)							
Pentadecanoic acid (15:0)	0.15	0.09	0.24	−0.0007	0.99	0.99	NS
Heptadecanoic acid (17:0)	−0.13	0.14	0.28	−0.06	0.32	0.48	NS
Palmitic acid (16:0)	0.23	0.01	0.06	0.01	0.86	0.94	NS
Stearic acid (18:0)	0.03	0.77	0.77	0.26	<0.0001	<0.001	0.04
Total SFAs ^c^	0.37	<0.0001	<0.001	0.27	<0.0001	<0.001	NS
*cis*-Monounsaturated fatty acids (MUFAs)							
Palmitoleic acid (16:1n-7,9)	0.20	0.03	0.12	0.20	0.001	0.006	NS
Oleic acid (18:1n-9)	−0.04	0.66	0.74	−0.10	0.11	0.25	NS
Total *cis*-MUFAs ^d^	−0.076	0.41	0.61	−0.09	0.13	0.26	NS
n-6 polyunsaturated fatty acids (PUFAs)							
Linoleic acid (18:2n-6)	−0.06	0.54	0.70	−0.12	0.06	0.16	NS
γ-Linolenic acid (18:3n-6)	0.05	0.56	0.70	0.24	0.0001	0.001	NS
Arachidonic acid (20:4n-6)	−0.14	0.12	0.26	0.05	0.40	0.52	NS
Total n-6 PUFAs ^e^	−0.11	0.25	0.42	−0.11	0.08	0.20	NS
Total long-chain n-6 PUFAs ^f^	−0.07	0.47	0.67	0.07	0.23	0.41	NS
*cis*-n-3 PUFAs							
α-Linolenic acid (18:3n-3)	0.19	0.04	0.12	−0.07	0.25	0.41	0.027
Eicosapentaenoic acid (EPA, 20:5n-3)	0.03	0.72	0.75	0.21	<0.001	0.004	NS
Docosahexaenoic acid (DHA, 22:6n-3)	−0.25	0.006	0.06	0.003	0.96	0.99	NS
Total n-3 PUFAs ^g^	−0.18	0.04	0.12	0.05	0.44	0.55	NS
Total long-chain n-3 PUFAs ^h^	−0.20	0.03	0.12	0.06	0.36	0.51	NS
Industrial *trans* fatty acids (iTFAs)							
Palmitelaidic acid (16:1n-9)	0.10	0.28	0.44	−0.14	0.03	0.10	NS
Elaidic acid (18:1n-9/12)	−0.18	0.04	0.12	−0.10	0.12	0.26	NS
Linoleic acid (18:2tt, ct, tc)	−0.06	0.52	0.70	−0.13	0.04	0.12	NS
α-Linolenic acid (18:3n-3ctt, ttc)	0.22	0.01	0.06	0.08	0.17	0.32	NS
Total iTFAs	−0.03	0.70	0.74	−0.14	0.02	0.07	NS
Ruminant *trans* fatty acids (rTFAs)							
Vaccenic acid (18:1n-7)	−0.24	0.008	0.06	−0.05	0.39	0.52	NS
Conjugated linoleic acids (CLA, 9c-11t; 10t,12c)	−0.04	0.67	0.74	−0.010	0.88	0.94	NS
Total rTFAs	−0.14	0.12	0.26	−0.04	0.52	0.62	NS
Ratio							
n-6 PUFAs/n-3 PUFAs	0.14	0.11	0.26	−0.07	0.28	0.44	NS
Long-chain n-6 PUFAs/long-chain n-3 PUFAs	0.12	0.19	0.36	−0.03	0.62	0.71	NS
Desaturation index_16_ (DI_16_, 16:1/16:0)	0.11	0.22	0.39	0.19	0.02	0.06	NS
Desaturation index_18_ (DI_18_, 18:1/18:0)	−0.03	0.71	0.75	−0.19	0.002	0.01	0.04

^a^ Models were adjusted for age, alcohol consumption, smoking, energy intake, education, physical activity, menopausal status in women, and batch of analysis. ^b^ Value for FDR correction. ^c^ Total SFA included 10:0, 12:0, 14:0, 15:0, 16:0, 17:0, 18:0, 20:0, 22:0, 24:0; ^d^ Total *cis*-MUFA included 14:1, 15:1, 16:1n-7,9, 17:1, 18:1n-5, 7, 9, 20:1, 22:1, 24:1; ^e^ Total n-6 PUFA included 18:2, 18:3, 20:2, 20:3, 20:4, 22:4, 22:5; ^f^ Total long-chain n-6 PUFA included 20:2, 20:3, 20:4, 22:4, 22:5; ^g^ Total n-3 PUFA included 18:3, 18:4, 20:4, 20:5, 22:5, 22:6; ^h^ Total long-chain n-3 PUFA included 20:4, 20:5, 22:5, 22:6; 18:2tt,ct,tc is a mixture of *trans*, *trans*, *cis*, *trans* and *trans*, *cis* isomers; 18:3n-3ctt, ttc is a mixture of *cis*, *trans*, *trans*, and trans, *trans*, *cis* isomers.
